# Epidemiology of Noise-Induced Tinnitus and the Attitudes and Beliefs towards Noise and Hearing Protection in Adolescents

**DOI:** 10.1371/journal.pone.0070297

**Published:** 2013-07-24

**Authors:** Annick Gilles, Guido Van Hal, Dirk De Ridder, Kristien Wouters, Paul Van de Heyning

**Affiliations:** 1 University Department of Otorhinolaryngology and Head & Neck Surgery, Antwerp University Hospital, Edegem, Belgium; 2 Faculty of Medicine, Campus Drie Eiken, Antwerp University, Wilrijk, Belgium; 3 Department of Epidemiology and Social Medicine, Medical Sociology and Health Policy, Antwerp University, Wilrijk, Belgium; 4 Tinnitus Research Initiative Centre (TRI), Antwerp University Hospital, Edegem, Belgium; 5 Department of Surgical Sciences, Dunedin School of Medicine, University of Otago, New Zealand; 6 Department Medical Management, Statistical Analysis, Antwerp University Hospital, Edegem, Belgium; University of Regensburg, Germany

## Abstract

**Background and objectives:**

Previous research showed an increase of noise-induced symptoms in adolescents. Permanent tinnitus as a consequence of loud music exposure is usually considered as noise-induced damage. The objective was to perform an epidemiological study in order to obtain prevalence data of permanent noise-induced tinnitus as well as temporary tinnitus following noise exposure in a young population. In addition the attitudes and beliefs towards noise and hearing protection were evaluated in order to explain the use/non-use of hearing protection in a young population.

**Methods:**

A questionnaire was completed by 3892 high school students (mean age: 16.64 years old, SD: 1.29 years). The prevalence of temporary and permanent tinnitus was assessed. In addition the ‘Youth Attitudes to Noise Scale’ and the ‘Beliefs About Hearing Protection and Hearing Loss’ were used in order to assess the attitudes and beliefs towards noise and hearing protection respectively.

**Results:**

The prevalence of temporary noise-induced tinnitus and permanent tinnitus in high school students was respectively 74.9% and 18.3%. An increasing prevalence of temporary tinnitus with age was present. Most students had a ‘neutral attitude’ towards loud music and the use of hearing protection was minimal (4.7%). The limited use of hearing protection is explained by a logistic regression analysis showing the relations between certain parameters and the use of hearing protection.

**Conclusions:**

Despite the very high prevalence of tinnitus in such a young population, the rate of hearing protection use and the knowledge about the risks of loud music is extremely low. Future preventive campaigns should focus more on tinnitus as a warning signal for noise-induced damage and emphasize that also temporary symptoms can result in permanent noise-induced damage.

## Introduction

Adolescents and young adults often expose themselves to loud music and excessive noise levels during social and music events [1]–[4]. Such excessive noise levels are often obtained in night clubs where levels between 104 and 112dB(A) can be measured [3]. Another source of leisure noise in the younger generation is personal listening devices (PLD’s) which many teenagers and young adults use at hazardous volume settings [5], [6]. Frequent leisure noise exposure louder than 90 dB(A) holds a significantly higher risk for the development of hearing problems [7], [8]. As a consequence, the younger generation showed an increment of hearing loss and noise-induced hearing symptoms such as tinnitus and hyperacusis over the last twenty years [9]–[12]. Tinnitus is defined as the perception of an auditory phantom sound in the form of ringing, buzzing, roaring or hissing in the absence of an external sound source [13] whereas hyperacusis is defined as a reduction of normal tolerance for everyday sounds. In the third National Health and Nutrition Examination Survey 12.5% of students, aged 6 to 19 years old, were identified with a noise-induced threshold shift characterized by a typical noise notch on the audiogram [10]. Other authors have confirmed audiometry-proven hearing damage in adolescents [14], [15].

Temporary noise-induced tinnitus (NIT) is also a common phenomenon in adolescents as reported prevalence numbers in previous studies vary between 45% and 77% [15]–[19]. Although most hearing symptoms such as tinnitus and hearing loss after loud music exposure have a temporary character, such symptoms are a clear sign of overexposure. The presence of temporary NIT after loud music exposure, even in the absence of hearing loss, is possibly accompanied by cochlear and/or neural damage which is not always perceived by the individual himself/herself nor measurable by a classical audiogram [20]. Hence, the experience of temporary NIT may be a relevant precursor for future symptoms such as permanent tinnitus or hyperacusis [20]–[22]. Frequent exposure to loud music at a young age can cause numerous adverse effects in a later stage such as the increase of vulnerability of the inner ear to aging [22]. In addition, noise-induced hearing loss (NIHL) can cause various problems such as a poorer quality of life related to reduced social interactions, isolation, a sense of exclusion, depression, and possibly impaired cognitive function [23].

In contrast to the high rate of hearing symptoms after loud music exposure and despite the fact that adolescents claim to be aware of the risks of loud music [24], [25], the use of hearing protection (HP) is rather limited. Various international studies evaluated the use of HP in different countries. A web-based survey in the US showed that 14% of the adolescents use HP in places where loud music is being played [16]. Conversely, in a Brazilian study only 1.6% reported to use HP [17]. Consequently, the suggestion can be made that the use of HP might be country-dependent. Therefore, Widen et al. (2006) performed a study in which the use of HP was compared between Swedish and US adolescents. In this study, Swedish adolescents were 12.8 times more likely to use HP when attending concerts compared to US youth (respectively 61.2% and 9.5% of the interviewed adolescents used HP). Analysis showed that attitudes towards noise and country explained 50% of the variance in the use of HP [26]. The authors suggested that the informational campaigns in Sweden emphasized the prevention of noise-induced damage by leisure noise to a higher extent than US campaigns which mainly focused on industrial noise exposure. Weichbold and Zorowka (2007) however, reported only a small increase in HP use from 0% to only 3.7% of the questioned adolescents after an intervention campaign suggesting that not all preventive hearing protection campaigns have effects on the behavior of adolescents concerning the protection of their hearing [27], [28]. The way in which HP use is questioned does also seem to be very important. When providing a yes/no question the prevalence of HP use lies much higher compared to the situation where more answer possibilities are provided (e.g. ‘never’, ‘sometimes’ or ‘always’). Therefore the latter approach seems to provide a more accurate estimate of HP use [29].

As mentioned earlier, the prevalence numbers of hearing symptoms after loud music exposure vary between studies. Therefore, the need for an epidemiological study rises. In order to understand adolescents’ behavior concerning the protection of their hearing, the attitudes and beliefs towards noise and HP must be assessed. While several studies analyzed the attitudes and beliefs of young people towards noise [26], [30], this has, to our knowledge, never been linked to the actual degree of HP use in a young population. The present study performed an epidemiological analysis in order to assess the prevalence of noise-induced hearing symptoms in a young population, with special attention for NIT as a symptom of overexposure to noise. In addition, the attitudes towards noise and HP were assessed and a model was built in order to explain the use of HP in adolescents and young adults. The relevance of attitudes and beliefs towards noise and hearing protection can be found in Ajzen’s Theory of Planned Behavior (TPB) [31]. The TPB is one of the most influential models for the prediction of human social behavior [32]. The TBP states that one’s attitude towards performing a specific behavior is a predictor of an intention. Intention is influenced by attitudes, subjective norms, and perceived behavioral control towards the behavior. Attitudes are regarded as beliefs about the outcome determined by positive or negative evaluation of self-performance of the particular behavior. A subjective norm is the extent to which an individual’s perception about the particular behavior is influenced by significant others (parents, peers, teachers, etc.) weighted by the compliance with such influence. Such a subjective norm might be the advice of peers to wear HP in noisy situations. Perceived behavioral control is an individual’s belief about the presence of factors that facilitate or impede the performance of the health-related behavior. Better insights into the thinking pattern and hearing protection habits of adolescents may provide helpful information for future preventive measures.

## Methods

### Ethics Committee

The principals of several high schools were contacted by phone with the suggestion to participate in the study. This approach was chosen because this allowed to provide sufficient information concerning the study and to answer all questions. After a positive verbal agreement, all participating schools were sent a written confirmation of participation by e-mail including a copy of the questionnaire. As the study is performed by the administering of a questionnaire, the high school principals were in this case considered as the caretakers of the minors. All questionnaires were administered during class. Students were not at all obliged to complete the questionnaire so the completion of the questionnaire was considered as a silent approval for participation. As such, an additional informed consent was not documented. The approach of the present study was approved by the IRB of the University Hospital Antwerp in May 2011.

### Participants

A cross sectional survey by means of a self-administered questionnaire was performed. Therefore a total of 4800 questionnaires were administered to students of fifteen randomly chosen Flemish high schools (age range: 14 to 18 years old; mean age: 16.64 years old, SD: 1.29 years). Students of all Flemish provinces and with all educational programs were represented in the sample. Table 1 shows the demographics of the participants as well as the age categories used for further analysis. A total of 83% (3991 out of 4800) of the questionnaires were completed, returned and were analyzed. Consequently, a drop-out of 17% was noted. The non-returned questionnaires were a result of absentees or students who did not finish or return the questionnaire.

**Table 1:** Distribution of male and female students among the age categories (absolute numbers).

Age (Years)	Female	Male	Total
14	38	27	65
15	470	402	872
16	428	407	835
17	568	448	1016
18	406	330	736
18+	125	193	318

### Questionnaire Structure

The questionnaire assessed following items: demographic and educational information, prevalence of permanent tinnitus and tinnitus characteristics, prevalence of temporary tinnitus and tinnitus characteristics, leisure noise exposure, attitudes towards noise and attitudes towards HP. All categories are explained in more detail in the following sections.

### Demographic and Educational Information

Students had to fill in their gender (male/female), year of birth and highest obtained educational diploma (all educational programs of Flanders were answer possibilities). Also the highest diploma obtained by their parents was questioned.

### Tinnitus: Prevalence and Characteristics

In the survey the term ‘tinnitus’ was replaced by a more laymen’s term (comparable to ‘ringing in the ears’ in the English language) as ‘tinnitus’ might be an unfamiliar term for adolescents. For our convenience the term ‘tinnitus’ is used throughout the manuscript instead of ‘ringing in the ears’.

Firstly, the prevalence of permanent tinnitus was evaluated by a yes/no question (‘Do you constantly perceive tinnitus?’). Secondly, the presence of temporary tinnitus after recreational noise exposure was evaluated by a Numeric Rating Scale (NRS) for loudness going from 0 (no tinnitus present) to 10 (extremely loud, cannot possibly be louder). For those students who already perceived a permanent tinnitus a worsening of tinnitus after loud music exposure was evaluated by use of a baseline-NRS (tinnitus intensity in normal condition) compared to a maximum-NRS (intensity of the loudest tinnitus experienced) after loud music exposure. A tinnitus loudness of 0 indicated the absence of NIT, while a tinnitus loudness >/1 indicated the presence of temporary NIT. The duration of temporary NIT or a temporary worsening of the permanent tinnitus was also assessed. Answer possibilities were: ‘less than thirty minutes’, ‘between thirty minutes and two hours’, ‘between two hours and six hours’, ‘between six hour and one day’, ‘more than one day’ and ‘not applicable’ in case if no tinnitus or no worsening of tinnitus was experienced. Furthermore, in case of temporary NIT, fear of permanent tinnitus was evaluated by questioning whether someone is afraid the tinnitus will not disappear (answer possibilities: always, often, sometimes, never). In a similar way it was evaluated whether one has the feeling the hearing decreases after noise exposure (answer possibilities: always, often, sometimes, never).

### Leisure Noise Exposure

For the evaluation of leisure noise exposure following situations had to be answered either on a ‘daily basis’, ‘weekly’, ‘monthly’, ‘yearly’ or ‘not applicable’: playing an instrument solo, playing an instrument in a band, listening to PLD’s, and discotheque attendance. The volume setting of PLD’s was assessed with a scale from 0% to 100% of the PLD capacity and music loudness in discotheques had to be rated as “too quiet”, “quiet”, “good”, “loud”, or “too loud”.

### Youth Attitudes to Noise Scale

The Youth Attitudes to Noise Scale (YANS) is an instrument designed by Widén & Erlandsson (2004) [33] in order to explore adolescents’ attitudes towards noise. Nineteen items are formulated in the form of statements and are measured on a five-point Likert scale going from ‘totally agree’ to ‘totally disagree’. The scale deals with different types of common sounds in adolescents’ environment which is categorized into four categories: 1) items dealing with attitudes towards noise in youth culture (e.g. sound levels in discotheques), 2) items dealing with attitudes towards the ability to concentrate in noisy environments, 3) items dealing with attitudes towards daily noises (e.g. traffic noise), 4) items dealing with attitudes towards the ability to influence the sound environment. Depending on the scores for the entire YANS as well as the scores on the different factors, a distinction can be made between a negative (lower quartile), a neutral (two middle quartiles) and a positive (upper quartile) attitude towards noise. A positive attitude towards noise implies that noise is regarded as something positive whereas a more negative attitude towards noise implies noise is seen as something dangerous or something that should be avoided. A neutral attitude in this case reflects a rather indifferent attitude towards noise meaning one does not care about or is unaware of the possible consequences of loud noises [30]. In the present study a validated Dutch version of the YANS was used [34]. For more information concerning the validity of the Dutch version of the YANS and the coding of the items, we refer the reader to Appendix S1.

### Beliefs About Hearing Protection and Hearing Loss

The ‘Beliefs About Hearing Protection and Hearing Loss’ (BAHPHL) is originally a 31-item questionnaire developed by the National Institute for Occupational Safety and Health [35]. The questionnaire was previously used in order to evaluate the attitudes towards HP and hearing loss (HL) in Swedish workers [36]. In the present study a validated Dutch version was used in which the items concerning occupational noise were omitted and the remaining items were adapted so the questionnaire was applicable to adolescents and contained 24 items in seven categories: 1) susceptibility to hearing loss, 2) severity of consequences of hearing loss, 3) benefits of preventive actions, 4) barriers to preventive actions, 5) behavioral intentions, 6) social norms and 7) self-efficacy [34]. The eighth category was omitted. Similar to the analysis of the YANS a positive, neutral and negative attitude towards HP and hearing loss can be distinguished. For more information concerning the validity of the Dutch version of the BAHPHL and the coding of the items, we refer the reader to Appendix S2.

### Statistical Analysis

For the statistical analysis a statistical Software package (SPSS 17.0, Inc., Chicago, IL, USA) was used in order to perform independent T-tests and Mann-Whitney U tests for the comparison of the prevalence of temporary and permanent tinnitus per age category and for the analysis of the scores on the YANS and BAHPHL. A step-wise logistic regression model was used to explain the use of HP in adolescents. Nineteen variables were put into the equation: Gender, age, smoking, education level, education level of the mother, education level of the father, temporary tinnitus after loud music exposure, NRS scores for temporary NIT and permanent tinnitus, persistence of NIT, fear of permanent tinnitus, subjective temporary hearing loss, total score on the YANS, total score on the BAHPHL, frequency discotheque visit, rating discotheque loudness, frequency music instrument playing, frequency use of PLD’s and volume settings of PLD’s. The level of statistical significance was defined as *p*<0.05.

## Results

### Tinnitus: Prevalence and Characteristics

**Figure 1:**
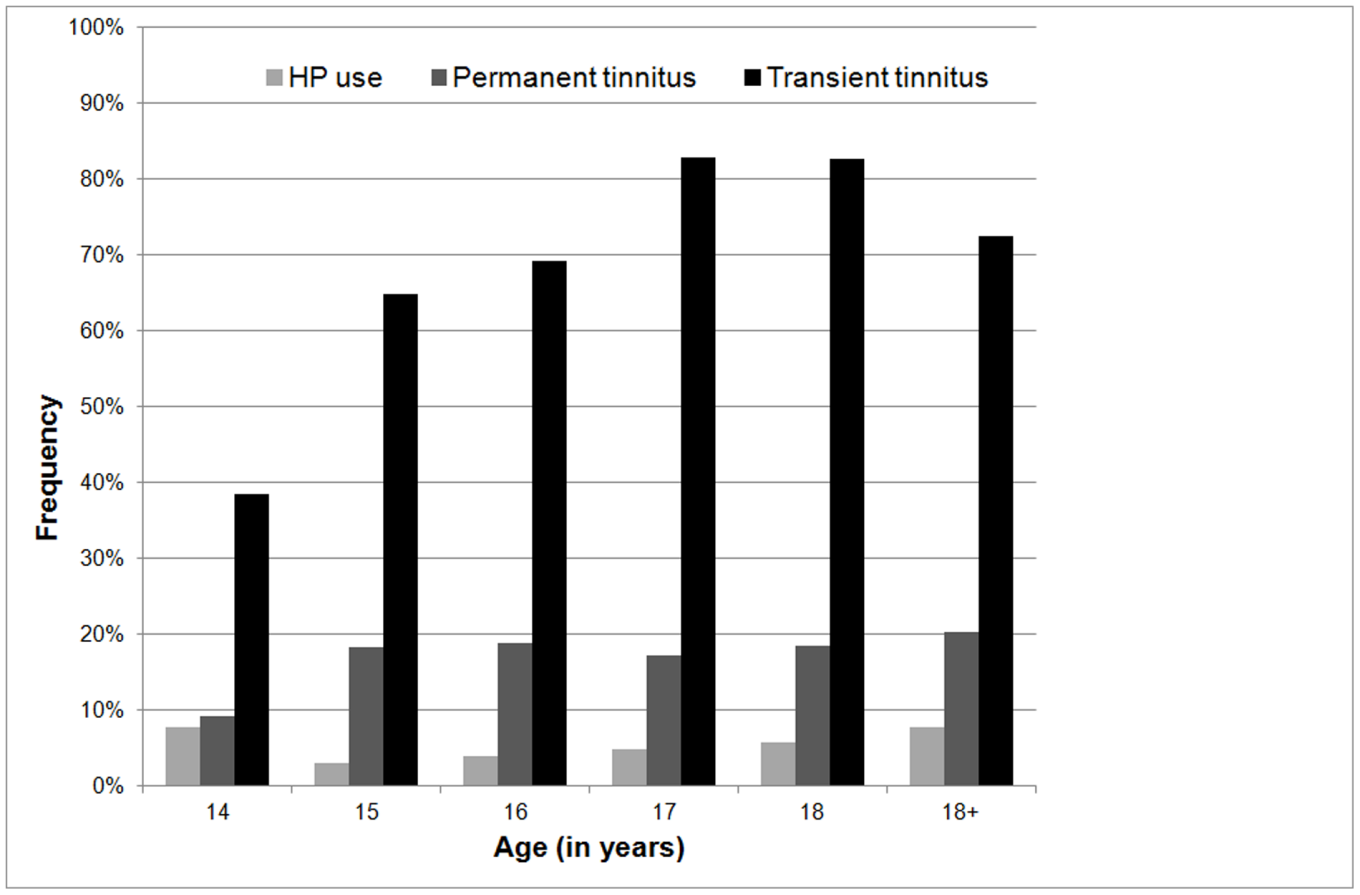
Tinnitus prevalence. Tinnitus prevalence (temporary as well as permanent) and HP use per age category.

Figure 1 illustrates the tinnitus prevalence per age category. 18.3% (confidence interval = 18.3% ±1.18%) of all students reported the experience of a permanent tinnitus in one or both ears. There were no significant differences between the age groups, with exception of the 14 year-olds who reported significantly less permanent tinnitus (9.2%, *p = *0,033) compared to the other age categories. Male respondents reported significantly more permanent tinnitus (20%) compared to female respondents (17%, *p* = 0.028). The overall prevalence of temporary NIT was 74.9% (confidence interval = 74.9% ±1.37%). In most cases tinnitus was perceived bilaterally as shown in table 2. A significant increase of temporary NIT with age was present in adolescents until 17 years old. Most students (75%) with permanent tinnitus rated loudness on the NRS with a score of 3 or less (mean score = 0.49±1.34). For temporary NIT, the tinnitus loudness was rated significantly higher (mean score = 2.96±2.53; p<0.001), with a score of 5 or less on the NRS for 76.4% of respondents. In 63.2% NIT or a temporary worsening of permanent tinnitus after noise exposure was present up to two hours after noise exposure. In approximately 10% of the respondents tinnitus persisted longer than two hours and in 3.5% tinnitus persisted for more than one day. Nevertheless, the majority (94.8%) of students did not fear permanent tinnitus. When asked about the perception of hearing loss after noise exposure, 39.1% of the respondents reported to sometimes experience a temporary subjective NIHL and 11.4% often to always experience a temporary NIHL. Despite the frequently experienced symptoms of hearing damage, HP was only used by 4.7% of the students, as also illustrated in figure 1.

**Table 2:** Prevalence of unilateral and bilateral permanent/temporary tinnitus.

	Total	Unilateral	Bilateral
**Permanent tinnitus**	18.3%	3.6%	14.7%
**Temporary tinnitus**	74.9%	2.1%	72.8%

### Leisure Noise Exposure

29.7% of students reported to play a musical instrument of which 13.5% on a daily basis. Moreover, 13.5% played in a band, mostly on a weekly basis (8.3%) and only few daily (0.9%). PLD use did not differ between age groups with up to 60% of all students using PLD’s on a daily basis for 30 minutes to one hour in 41.7% of the cases. Furthermore, 35.4% sets the volume settings up to 80% or more of the total PLD capacity.

The evaluation of party behavior showed a significant increase of discotheque attendance with age as 4.6% of the 14 year old and 30.4% of the 18 year olds visited discotheques on a daily to weekly basis. When asked to rate music loudness in discotheques, 45.1% reported it to be ‘too loud’, 43.9% considered the levels as ‘loud’ and 10.9% reported them to be quiet or sufficient.

### YANS and BAHPHL

Table 3 shows the mean values and standard deviations for the entire YANS as well as the separate factors. In accordance with Widen et al. (2009) [30] the YANS was divided into three categories by use of the quartiles of the sum of the scale representing a positive (scores 0–2.78), neutral (scores 2.79–3.41) or negative (scores 3.42–5) attitude towards noise. 27.6% of all students held a negative attitude, meaning noise is regarded as something dangerous. On the opposite, 26.1% held a positive attitude where noise is not seen as a threat. The remaining 46.3% held a neutral attitude towards noise. The attitude towards noise in relation to the use of HP was investigated. Students with a negative attitude towards noise used HP significantly more (p<0.001) than students with a neutral or positive attitude, despite the fact that the overall use of HP was very low (4.7%). No gender differences were found.

**Table 3:** Overview of the scores on the entire YANS and the four factors.

		N items	Mean	SD	Negativeattitudes	Neutralatittudes	Positiveattitudes
Factor 1	Youth culture	8	3.26	0.76	0–2.75	2.76–3.74	3.75–5.00
Factor 2	Concentration	3	2.80	0.76	0–2.33	2.34–3.32	3.33–5.00
Factor 3	Daily noises	4	3.33	0.80	0–2.75	2.76–3.99	4.00–5.00
Factor 4	Intent to influence	4	2.78	0.69	0–2.25	2.26–3.24	3.25–5.00
Entire YANS			3.10	0.49	0–2.50	2.51–3.24	3.25–5.00

Table 4 shows the mean values and standard deviations for the entire BAHPHL as well as the separate factors. The score on the entire BAHPHL did not show any significant changes with age nor with gender. Similar to the YANS, the group could be divided in a group with negative (scores 0–2.46), neutral (scores 2.47–3.03) and positive (scores 3.04–5.00) attitudes and beliefs towards noise and hearing loss. It was found that students with negative attitudes also used HP significantly more (p<0.001) compared to those with a neutral or positive attitude.

**Table 4:** Overview of the scores on the entire BAHPHL and the seven factors.

		N items	Mean	SD	Negativeattitude	Neutralattitude	Positiveattitude
Factor 1	Susceptibility to HL	6	2.42	0.68	0–2.00	2.01–2.82	2.83–5.00
Factor 2	Severity of consequences of HL	3	2.11	0.78	0–1.33	1.34–2.66	2.67–5.00
Factor 3	Benefits of preventive actions	3	2.23	0.73	0–1.67	1.68–2.66	2.67–5.00
Factor 4	Barriers to preventive actions	4	3.32	0.74	0–3.00	3.01–3.74	3.75–5.00
Factor 5	Behavioral intentions	3	3.32	0.97	0–2.67	2.68–3.99	4.00–5.00
Factor 6	Social norms	2	3.40	0.90	0–3.00	3.01–3.99	4.00–5.00
Factor 7	Self-efficacy	3	2.82	0.79	0–2.33	2.34–3.32	3.33–5.00
Entire BAHPHL			2.74	0.45	0–2.46	2.47–3.03	3.04–5.00

### Logistic Regression Model

A logistic regression model was used to determine which factors are involved when it comes to the use of HP when exposed to loud music. Seven variables out of the nineteen showed significant influence on the use of HP: gender, fear of permanent tinnitus, temporary tinnitus after loud music, rating discotheque loudness, permanent tinnitus and the score on the BAHPHL. The results of the logistic regression model are shown in table 5. The model shows that male adolescents were more likely to wear HP. Those who were afraid of the development of permanent tinnitus were also more inclined to use HP. The score on the BAHPHL was also a highly significant parameter for HP use (*p*<0.001). A trend for better usage of HP in adolescents with permanent tinnitus was observed, however the influence was only marginal significant (*p = *0,06). Rating the discotheque loudness as ‘too loud’ had positive influences on the use of HP. Surprisingly, the students experiencing temporary tinnitus used HP to a lesser extent (*p = *0004).

**Table 5:** Logistic regression model: explaining the use of hearing protection (n.a.: not applicable).

Variable	Reference	B-value	Odds ratio (OR)	95% C.I. for OR: lower	95% C.I. for OR: upper	*p*-value
**Gender: Male**	Female	0.50	1.65	1.07	2.54	0.023
**Fear of persistence of temporary tinnitus**	No fear	1.23	3.43	1.85	6.37	<0.001
**Temporary tinnitus**	Absence	−0.81	0.44	0.26	0.77	0.004
**Rating discotheque loudness**	‘too loud’					0.005
**- quiet**		0.91	2.48	1.24	4.99	0.011
**- loud**		−0.27	0.76	0.48	1.21	0.251
**Score BAHPHL**	n.a.	−0.12	0.89	0.87	0.91	<0.001
**Age**	n.a.	0.01	1.01	0.83	1.23	0.94
**Permanent tinnitus**	Absence	−7.65	<0.001	<0.001	1.37	0.060
Age*permanent tinnitus	n.a.	0.45	1.57	0.99	2.48	0.052

Originally nineteen items were put into the equation: Gender, age, smoking, education level, education level of the mother, education level of the father, temporary tinnitus after loud music, NRS score temporary NIT and permanent tinnitus, persistence of NIT, fear of permanent tinnitus, subjective temporary hearing loss, score on the YANS, score on the BAHPHL, frequency discotheque visit, rating discotheque loudness, frequency music instrument playing, frequency use of PLD’s and volume settings of PLD’s. The current table shows the variables that yielded statistical significance. Nagelkerke R^2^ was 0.3 meaning 30% of the variance was explained by the current model.

## Discussion and Conclusions

A significant number of the study population (18.3%) reported permanent tinnitus in one or both ears. The prevalence of permanent NIT in this adolescent population was threefold the prevalence found in the study of Widen et al (2004) [33], who found a prevalence of 8.7% in a population of 1285 young individuals between 13 an 19 years old. This while the group was quite similar to the respondents of the present study according to age, and the questioning of permanent tinnitus also happened in a similar way in both studies (yes-no question). However, the present study provided a NRS additionally to the yes-no question. It is possible that respondents might only confirm the presence of tinnitus in the case of a relevant tinnitus percept (NRS >2) when a yes-no question is presented. In the present study also respondents with a NRS of 1 were taken into account. Furthermore, as the authors attempted to assess the prevalence of NIT and permanent tinnitus in adolescents, a NRS loudness was deliberately chosen over a NRS distress. The reason lies within the fact that a subject’s scores for tinnitus loudness and tinnitus distress does not necessarily correlate. A score for tinnitus loudness is therefore more reliable as a marker for the presence of tinnitus.

An overall prevalence of 74.9% of temporary NIT was observed. Such a prevalence of temporary NIT is consistent with previous studies [15]–[17], [19]. However, the present study showed, for the first time, an age dependent symptomatology as a significant increase in temporary NIT with age going from 38.5% in 14-year-olds to 82.7% in 18-year-olds was revealed. The question arises whether this increase is due to the increase in social noise exposure. The frequency of PLD use and volume settings did not differ between age groups. Therefore, the increase of NIT may be related to the increased rate of discotheque attendance in the older adolescents. Music levels in discotheques are typically in the range of >105 dB. Previous research learned that significant social noise exposure (>97 dB) triples the report of NIT [37] and frequent PLD users are four times more likely to listen to high-volume music than infrequent users [5], [38]–[41]. In the present study approximately one third of the respondents regularly listened to PLD’s at hazardous noise levels (>80% of the capacity) so an additive effect of years of PLD use at excessive noise levels should be taken into account.

The underlying construct of the TPB (attitudes, social norms and perceived behavioral control) were partly followed in the present study. The attitudes towards noise were assessed by the YANS whereas subjective norms and perceived behavioral control can be found in the BAHPHL. Although both scales have intrinsic distinct subcomponents, they are all part of the construct of a certain belief and certain attitudes, in this case beliefs and attitudes concerning noise, the development of hearing loss and the use of hearing protection. As it is believed that beliefs and attitudes are responsible for the eventual ‘actions’ (e.g. the actual use of hearing protection) we were mainly interested in the total scores of the YANS and BAHPHL because it is the total construct that may underlie the actions and not the subcomponents of the construct. While the YANS focuses more on the attitudes towards noise, the BAHPHL deals particularly with HP use. The experience of a temporary NIHL in about 50% of the cases seems to positively affect the score on the BAHPHL. In the logistic regression model the total BAHPHL score indeed was a highly significant (*p*<0.001) parameter. The lower the score on the BAHPHL, the more positive one was towards HP and the more HP was used. These findings confirm the findings of a recent study by Widén et al. (2013) who suggested that norms regarding HP use play a greater role than the attitudes towards HP and noise [29]. In the present study the YANS (which is a way of measuring the ‘attitudes’) did not yield statistical significance in the logistic regression and therefore does not explain any of the variance for the use of HP. The BAHPHL on the other hand, which includes factors such as ‘social norms’ and ‘barriers to preventive actions’, turned out as a very important parameter in the explaining of the variance for HP usage.

An earlier study on risk behavior and noise exposure among adolescents found that women and men behave identically concerning HP, although women judge risk situations more dangerous than men in general [42]. The present study did not find any significant gender differences concerning attitudes (YANS and BAHPHL) towards noise. However, male respondents were more likely to use HP than female respondents. A possible explanation lies in the fact that male students in the study population experience permanent tinnitus more often and therefore are more likely to protect their hearing from now on.

Personal rating of discotheque levels influences HP use as those rating the intensity levels at discotheques as ‘too loud’, intend to more often use HP. Considering that mean sound levels in discotheques vary between 104 and 112 dB [3], occupational safety standards are exceeded by far when visiting discotheques [35]. In our study population, 45% of the respondents consider discotheque levels as ‘*too loud’*, 44% claimed it to be ‘*loud’* and barely 11% rated the levels as ‘*sufficient’ or ‘quiet’*. Similar results were previously obtained by Mercier and Hohmann (2002) who concluded that the excessive sound levels at many music events are not demanded nor required by most young people. In addition, it has been found that people would not visit night clubs any less if the intensity levels would be lowered [27].

Fear of the persistence of tinnitus was also a good predictor for HP use as those fearing tinnitus were more inclined to use HP. In addition, although not significant, permanent tinnitus also seemed to be a motivational factor for HP use. Temporary NIT however, surprisingly, did not raise HP use. A possible explanation is that those students who do not use HP will be more prawn to the experience of temporary tinnitus. Secondly, is it plausible that as students are quite familiar with temporary symptoms, they do not find it necessary to take precautions because of the transient characteristic. In a previous study, young individuals were questioned concerning whether which hearing symptoms apply when noise is too loud and possibly damaging [43]. Tinnitus (‘ringing ears’) was only considered as a relevant symptom by approximately 15%. Such findings confirm the ignorance of young individuals concerning hearing symptoms after loud music exposure. Consequently, previous studies have shown the limited effects of preventive campaigns on adolescents [27], [28].

The authors like to point out that, up until now, most preventive campaigns mainly focused on the development of hearing loss as a consequence of frequent exposure to loud music. As a temporary threshold shift or even a mild permanent threshold shift as a consequence of noise exposure, is often not noticed by adolescents, the focus of campaigns on the development of hearing loss might be a less effective approach. As the present study shows a very high prevalence of tinnitus (temporary as well as permanent) in young people we suggest that preventive campaigns should focus more on tinnitus as a warning signal for noise-induced damage. In addition, the fact that also temporary tinnitus or other temporary noise-induced symptoms do not necessarily exclude permanent cochlear or neural damage and that through accumulative noise damage temporary symptoms may evolve into permanent symptoms, should be underlined. In the modern society, where social network sites and smartphones have become so important into the lives of young people, campaigns should use these communication modes in order to achieve adolescents’ attention. In addition, more focus should go to a more interactive way of communicating to make adolescents more aware of the risks of loud music exposure. The authors believe that the personal experience with noise-induced symptoms (e.g.: walking around for a week with an mp3-player constantly playing a high-pitched pure tone) is a fast and effective way to rapidly change the beliefs and attitudes and therefore the actions of adolescents. Furthermore, the authors suggest that preventive campaigns should also focus on students younger than 14 years old. As the results have shown an increase of attending social events with loud music and therefore an increase of hearing problems from this age on, educational programs should take place in advance as attitudes and thus beliefs and therefore behaviors have already been formed prior to this age. A better understanding of noise-induced symptoms may result in an increase of HP in a younger population and may prevent permanent hearing loss and tinnitus due to recreational noise exposure.

## Supporting Information

Appendix S1 **Youth Attitudes to Noise Scale (YANS).** Information concerning the YANS questionnaire and the validation of the Dutch YANS including Cronbach’s alpha scores.
(DOCX)

Appendix S2 **Beliefs About Hearing Protection and Hearing loss (BAHPHL).** Information concerning the BAHPHL questionnaire and the validation of the Dutch BAHPHL including Cronbach’s alpha scores.
(DOCX)
