# Supplementary material for: Epidemiology of Noise-Induced Tinnitus and the Attitudes and Beliefs towards Noise and Hearing Protection in Adolescents
Source: PLoS One. 2013 Jul 24;8(7):e70297. doi: 10.1371/journal.pone.0070297 (PMC3722160; doi:10.1371/journal.pone.0070297)
Supplement: Appendix S1 — Youth Attitudes to Noise Scale (YANS). Information concerning the YANS questionnaire and the validation of the Dutch YANS including Cronbach’s alpha scores. (DOCX) [file pone.0070297.s001.docx]

**Appendix 1: Youth Attitudes to Noise Scale**

The Youth Attitudes to Noise Scale is a scale which consists of 19 items, developed by Widén et al. (2004) in an attempt to explore adolescents’ attitudes towards noise. The items of the YANS are presented below in table 1 (English version). All items are scores on a five-point Likert scale going from “totally agree” (coded as 5) to “totally disagree” (coded as 1) and items 1, 3, 4, 7, 8, 12, 13, 15, 18 and 19 are inversely coded. The scale is intended to explore adolescent’s attitudes towards noise. Therefore, the scale deals with different types of common sounds in adolescent’s environment. The YANS can be subdivided into four factors: 1) items dealing with attitudes towards noise associated with elements of youth culture e.g. sound levels in discotheques (items 1, 4, 6, 9, 10, 12, 15 and 18); 2) items dealing with attitudes towards the ability to concentrate in noisy situations (items 2, 5 and 8); 3) items dealing with attitudes towards daily noises e.g. traffic noise (items 11, 14, 16 and 17) and 4) items dealing with attitudes towards influencing the sound environment (items 3, 7, 13 and 19). Depending on the scores for the entire YANS as well as the scores on the different factors, a distinction can be made between a negative (lower quartile), a neutral (two middle quartiles) and a positive (upper quartile) attitude towards noise. A negative attitude towards noise means that noise is seen as something harmful, as something “you would want to avoid”, whereas a positive attitude towards noise means that noise is not seen as something dangerous. A neutral attitude in this case reflects a rather indifferent attitude towards noise meaning one does not care or is unaware of the possible consequences of loud noises.

| Item 1 | I think that the sound level at discos, dances, rock concerts and sporting events, in general, is too loud. |
| --- | --- |
| Item 2 | Listening to music while doing homework helps me concentrate. |
| Item 3 | I am prepared to do something to make the school environment quieter. |
| Item 4 | I consider leaving a disco, rock concert, dance or sporting event if the sound level is too loud. |
| Item 5 | I can concentrate even if there are many different sounds around me. |
| Item 6 | I think it is unnecessary to use earplugs when I am at a disco, rock concert, dance or sporting event. |
| Item 7 | It is important for me to make my sounds environment more comfortable. |
| Item 8 | I don’t like when it is quiet around me. |
| Item 9 | The sound level at discos, dances, rock concerts or sporting events is not a problem. |
| Item 10 | Noise and loud sounds are natural parts of our society. |
| Item 11 | Traffic noise is not disturbing to me. |
| Item 12 | The sound level should be lowered at discos, rock concerts, dances or sporting events. |
| Item 13 | I think it should be quiet and calm in the classroom. |
| Item 14 | Sounds from fans, refrigerators, computers, etc., do not disturb me. |
| Item 15 | I am prepared to give up activities where the sound level is too loud. |
| Item 16 | The sound level at my school is comfortable. |
| Item 17 | It is easy for me to ignore traffic noise. |
| Item 18 | There should be more rules or regulations for the sounds levels in society. |
| Item 19 | When I cannot get rid of sounds that bother me, I feel helpless. |

*Table 1: Original English version of the 19 items of the YANS.*

Keppler et al. (2010) provided the validated Dutch version of the YANS, which was used in the present study, of which Cronbach’s alpha scores are listed in table 2.

|  | Factor 1 | Factor 2 | Factor 3 | Factor 4 | Entire YANS |
| --- | --- | --- | --- | --- | --- |
| Cronbach’s alpha | 0.73 | 0.44 | 0.60 | 0.27 | 0.71 |

*Table 2: Cronbach’s alpha scores for the entire YANS and the sub factors for the Dutch version of the YANS*.
