# Supplementary material for: Epidemiology of Noise-Induced Tinnitus and the Attitudes and Beliefs towards Noise and Hearing Protection in Adolescents
Source: PLoS One. 2013 Jul 24;8(7):e70297. doi: 10.1371/journal.pone.0070297 (PMC3722160; doi:10.1371/journal.pone.0070297)
Supplement: Appendix S2 — Beliefs About Hearing Protection and Hearing loss (BAHPHL). Information concerning the BAHPHL questionnaire and the validation of the Dutch BAHPHL including Cronbach’s alpha scores. (DOCX) [file pone.0070297.s002.docx]

**Appendix 2: Beliefs About Hearing Protection and Hearing Loss scale**

The Beliefs About Hearing Protection and Hearing Loss (BAHPHL) scale was developed by the US National Institute for Occupational Safety and Health (NIOSH) in order to assess the beliefs concerning hearing loss impact and the attitudes towards hearing protection (NIOSH contract no. 211-93-006). The scale is originally intended for industrial workers and contains 31 items (table 3) scored on a five-point Likert scale going from “totally agree” (coded as 1) to “totally disagree” (coded as 5). The BAHPHL contains eight subscales: 1) perceived susceptibility to hearing loss; 2) perceived severity of consequences of hearing loss; 3) perceived benefits of preventive action; 4) perceived benefits of preventive action – comfort; 5) perceived barriers to preventive action – important sounds muffled; 6) behavioral intentions (future, present and past behaviors); 7) social norms; and 8) self-efficacy. Similar to the YANS, scores in the lower quartile represent a negative attitude towards noise, scores in the middle two quartiles a neutral attitude and scores in the upper quartile a positive attitude towards noise.

| Item 1 | I think earmuffs put too much pressure on my ears. |
| --- | --- |
| Item 2 | I believe I know how to fit and wear earplugs. |
| Item 3 | I do not intend to wear hearing protectors when I am around loud tools or equipment. |
| Item 4 | Most of my co-workers wear hearing protectors when they work around loud noise. |
| Item 5 | I think I can work around louse noise without it hurting my hearing. |
| Item 6 | I think wearing hearing protectors every time I am working in loud noise is important. |
| Item 7 | I think earmuffs make my head sweat too much. |
| Item 8 | I wear hearing protectors whenever I work around loud noise. |
| Item 9 | Hearing protectors are uncomfortable to wear. |
| Item 10 | My co-workers don’t wear hearing protectors when they work in loud noise. |
| Item 11 | I’m not sure how to tell when earplugs need to be replaced. |
| Item 12 | Losing my hearing would make it hard for people to talk to me. |
| Item 13 | I believe my ears can eventually ‘get toughened’ to noise, so they are less likely to be damaged by it. |
| Item 14 | I know when I should use hearing protectors. |
| Item 15 | I think it will be hard to hear warning signals( like back-up beeps) if I am wearing hearing protectors. |
| Item 16 | I believe exposure to loud noise can hurt my hearing. |
| Item 17 | I am convinced I can prevent hearing loss by wearing hearing protectors whenever I work in loud noise. |
| Item 18 | I think my hearing is being hurt by exposure to loud noise at work. |
| Item 19 | Hearing protectors limit my ability to hear problems on the job site. |
| Item 20 | I don’t think it would be such a big handicap to lose part of my hearing. |
| Item 21 | If I wear hearing protection, I can protect my hearing. |
| Item 22 | I know how to tell when an earmuff needs to be replaces. |
| Item 23 | Wearing hearing protectors is annoying. |
| Item 24 | Most of my co-workers think it is a good idea to wear hearing protectors in hazardous noise. |
| Item 25 | If co-workers asked me, I would be able to help them wear hearing protectors correctly. |
| Item 26 | I don’t think I have to wear hearing protectors every time I am working in noise. |
| Item 27 | I can’t hear problems with my tools and machinery if I wear hearing protectors. |
| Item 28 | I believe that daily exposure to loud machinery and tools will eventually damage my hearing. |
| Item 29 | I think it would be a big problem if I lost my hearing. |
| Item 30 | I plan to wear hearing protection when I work near loud noises. |
| Item 31 | On my current job, I seldom wear hearing protectors when I work around loud noises. |

*Table 3: Original English version of 31 items of the BAHPHL.*

Keppler et al. (2010) provided a Dutch validated version of the BAHPHL. As the intention was to assess the beliefs and attitudes regarding the risks of hearing loss and the impact on hearing protection use of adolescents when exposed to recreational noise, some of the items of the original BAHPHL were omitted or altered to make them applicable to adolescents. As a result the Dutch version contains 24 items instead of 31 in seven content categories instead of eight: 1) susceptibility to hearing loss (items 4, 11, 13, 15, 21 and 22); 2) severity of consequences of hearing loss (items 10, 17 and 23); 3) benefits of preventive action (items 5, 14 and 18); 4) barriers to preventive action (items 1, 7, 16 and 19); 5) behavioral intentions (items 3, 6 and 24); 6) social norms (items 8 and 20); and self-efficacy (items 2, 9 and 12). An inverse coding was applied on items 1, 3, 4, 7, 8, 9, 11, 16, 17, 19 and 21 (“totally agree” = 5; “totally disagree” = 1). An English version of the adapted Dutch BAHPHL is provided in table 4.

| Item 1 | I think earmuffs put too much pressure on my ears. |
| --- | --- |
| Item 2 | I believe I know how to fit and wear earplugs. |
| Item 3 | I do not intend to wear hearing protectors when I am in loud environments. |
| Item 4 | I think I can stay in loud environments without it hurting my hearing. |
| Item 5 | I think wearing hearing protectors every time I am in loud environments is important. |
| Item 6 | I wear hearing protectors whenever I am in loud environments. |
| Item 7 | Hearing protectors are uncomfortable to wear. |
| Item 8 | My friends don’t wear hearing protectors. |
| Item 9 | I am not sure how to tell when earplugs need to be replaced. |
| Item 10 | Loosing my hearing would make it hard for people to talk to me. |
| Item 11 | I believe that my ears eventually ‘get toughened’ to noise, so they are less likely to be damaged by it. |
| Item 12 | I know when I should use hearing protectors. |
| Item 13 | I believe exposure to loud noise can hurt my hearing. |
| Item 14 | I am convinced I can prevent hearing loss by wearing hearing protectors whenever I am in loud environments. |
| Item 15 | I think my hearing is being hurt by exposure to loud noise. |
| Item 16 | Hearing protectors limit my ability to communicate with others. |
| Item 17 | I don’t think it would be such a big handicap to lose part of my hearing. |
| Item 18 | If I wear hearing protection, I can protect my hearing. |
| Item 19 | Wearing hearing protectors is annoying. |
| Item 20 | My friends think it is a good idea to wear hearing protectors in hazardous noise. |
| Item 21 | I don’t think I have to wear hearing protectors every time I am in loud environments. |
| Item 22 | I believe that daily exposure to loud noise will eventually damage my hearing. |
| Item 23 | I think it would be a big problem if I lost my hearing. |
| Item 24 | I plan to wear hearing protection when I am in loud environments. |

*Table 4: Translation of the Dutch version of the BAHPHL containing 24 items.*

Cronbach’s alpha scores were not provided in the dissertation of Keppler et al. (2010). Therefore the Cronbach’s alpha scores were calculated for the BAHPHL for the present study and presented in table 5.

|  | Factor 1 | Factor 2 | Factor 3 | Factor 4 | Factor 5 | Factor 6 | Factor 7 | Entire BAHPHL |
| --- | --- | --- | --- | --- | --- | --- | --- | --- |
| Cronbach’s alpha | 0.67 | 0.75 | 0.69 | 0.73 | 0.67 | 0.72 | 0.74 | 0.65 |

*Table 5: Cronbach’s alpha scores for the entire BAHPHL and the sub factors for the Dutch version of the BAHPHL*.
